# A new species of *Decimiana* Uvarov (Insecta, Mantodea, Acanthopidae) from Brazil, with remarks on the distribution of *Decimiana bolivari* (Chopard) 

**DOI:** 10.3897/zookeys.236.3477

**Published:** 2012-10-02

**Authors:** Eliomar da Cruz Menezes, Freddy Bravo

**Affiliations:** 1 Laboratório de Sistemática de Insetos, LABIO, Departamento de Ciências Biológicas, Universidade Estadual de Feira de Santana, Av. Transnordestina, S/N, Novo Horizonte, 44.036-900, Feira de Santana, Bahia, Brazil

**Keywords:** Dictyoptera, Neotropical region, Bahia, Chapada Diamantina, Caatinga

## Abstract

*Decimiana* Uvarov is a Neotropical genus of Mantodea with five South American species, three of them known from Brazil: *Decimiana tessellata* (Charpentier); *Decimiana clavata* Ippolito & Lombardo; and *Decimiana bolivari* (Chopard). A fourth species from Brazil is described and new records of *Decimiana bolivari* (Chopard) from Brazil are presented and its distribution discussed.

## Introduction

The *Decimiana* name was proposed by Uvarov (1940) to replace the genus name *Decimia*, designated by Stål (1877), due to homonymy with a genus of Lepidoptera (Travassos 1945). This genus was created in monotype to *Acanthops tessellata* Charpentier, 1841 (Travassos 1945; Lombardo 2000).

*Decimiana tessellata* (Charpentier, 1841), was designated based on Seba’s illustration (1765: 75, Fig. 11) (Travassos 1945). Illustration which historically were also attributed the representation of species *Acanthops falcataria* (Goeze, 1778) [=*Mantis falcataria* Goeze]and *Mantis bisubulata* Goeze, 1778 considered a junior synonym of *Decimiana tessellata* despite the name priority (Travassos 1945; Lombardo 2000). Lombardo (2000) to analyze the same Seba’s illustration concludes that represent *Decimiana tessellata*.

Lombardo (2000) in his revision of the genus includes *Decimiana bolivari* (Chopard, 1916) [=*Acanthops bolivari* Chopard], *Decimiana rehni* (Chopard, 1913) [=*Plesiacanthops rehni* Chopard], transferred from other genera, and described *Decimiana hebardi* Lombardo. Later Ippolito and Lombardo (2004) described *Decimiana clavata*, a species know only from the holotype male. In his revision Lombardo (2000) affirms describe the first time the males of *Decimiana bolivari*, species that had been described based on females. However, in a study of morphologic stamp, Heitzmann-Fontenelle (1964) describes and figure the male and female of *Acanthops erosula* Stål, 1877, which corresponds in reality to *Decimiana bolivari* (Lombardo and Ippolito 2004).

The genus *Decimiana* has Neotropical distribution and their five species are recorded only in South America (Lombardo 2000, Ippolito and Lombardo 2004). Of which three species occurs in Brazil: *Decimiana bolivari* (Chopard) from Paraguay and Brazil (Bahia State, in the northeastern region, and Mato Grosso do Sul State, in the central-western region); *Decimiana clavata* Ippolito & Lombardo from Brazil (with no mention of a specific locality); *Decimiana hebardi* Lombardo from Bolivia; *Decimiana rehni* (Chopard) from Argentina and Paraguay; and *Decimiana tessellata* (Charpentier) from Paraguay and Brazil (Mato Grosso and Goiás states, central-western region) (Terra 1995, Lombardo 2000, Ippolito and Lombardo 2004).

In this contribution, we described a new species of Decimiana from Bahia State in the northeastern region of Brazil. Additionaly, we provide and discuss new distributional records for *Decimiana bolivari* from Brazil.

## Methods

All of the specimens were collected using a light trap. To study the male genitalia, the abdomens of the specimens were detached behind the eighth segment and treated according to the protocols of Cumming (1992). The male genitalia were stored in plastic microvials containing glycerin and pinned with their corresponding specimen. The terminology for external morphological follows Terra (1995), whereas genitalia terminology is based on Cerdá (1993). The studied specimens were deposited in the Coleção Entomológica Professor Johann Becker do Museu de Zoologia da Universidade Estadual de Feira de Santana (MZUEFS), Feira de Santana, Bahia State, Brazil; the Museu de Zoologia da Universidade Federal da Bahia (UFBA), Salvador, Bahia State, Brazil and Centro de Pequisa Gonçalo Moniz da Fundação Oswaldo Cruz (FIOCRUZ), Salvador, Bahia State, Brazil.

## Taxonomy

The new species have the following diagnostics characteristics of the genus: presence of compound eyes with conical tubercle; costal margin of the mesothoracic wings with slight concavity; posterior wings with black stripes on cross veins; mid and hind legs without lobes, and anterior process of the ventral lamina developed (*sensu* Lombardo 2000).

### Decimiana elliptica sp. n.

urn:lsid:zoobank.org:act:7F0879A2-1852-4ACE-8518-F7B4475EF1F2

http://species-id.net/wiki/Decimiana_elliptica

**Figure 1.**
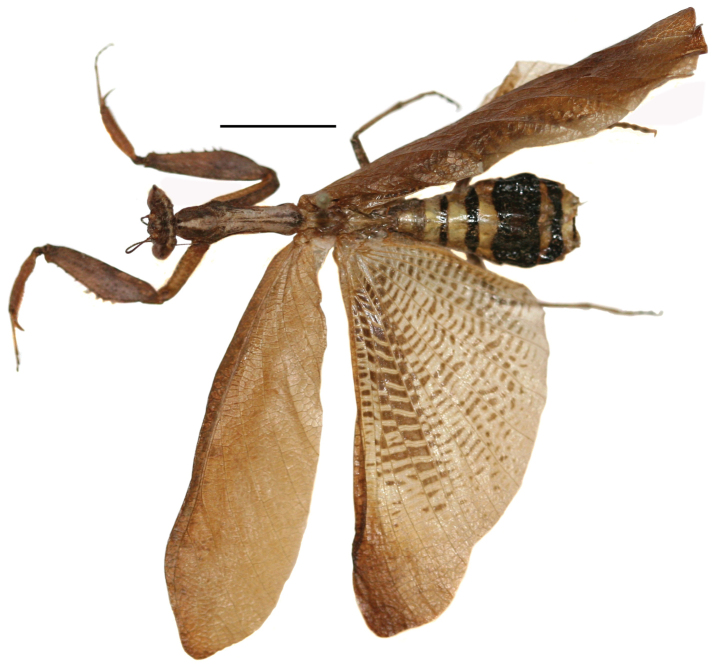
*Decimiana elliptica* sp. n., holotype, dorsal habitus. Scale bar = 10.00mm.

Figures 1 [Fig F2] –3

#### Type material.

Holotype male: BRASIL, Bahia, Palmeiras, Posto do Pai Inácio, 12°27.00"S, 41°28.00"W, ca. 900 m a.s.l. 09.XII.2007, Bravo, F., Zacca, T., Silva-Neto, A., Resende, J., & Almeida, C. col., (MZUEFS #42169). Paratype male: BRASIL, Bahia, Mucugê, Chapada Diamantina, Parque Municipal de Mucugê, 30.I.2011, light trap, Mahlmann, T. & Hipólito cols. (UFBA).

#### Etymology.

The name makes reference to the shape the anterior lobe of the left dorsal phallomere.

#### Diagnosis.

Compound eyes conical with apical tubercle; mesothoracid wings opaque, brown, with costal margin slightly concave; posterior wings with black bands between the crossveins; anterior process of the left dorsal phallomere with distal sclerotized lobe elliptical.

#### Description male.

Body stout, brown (Fig. 1), length 38.64–42.68 mm from head to subgenital plate.

**Figure 2. F2:**
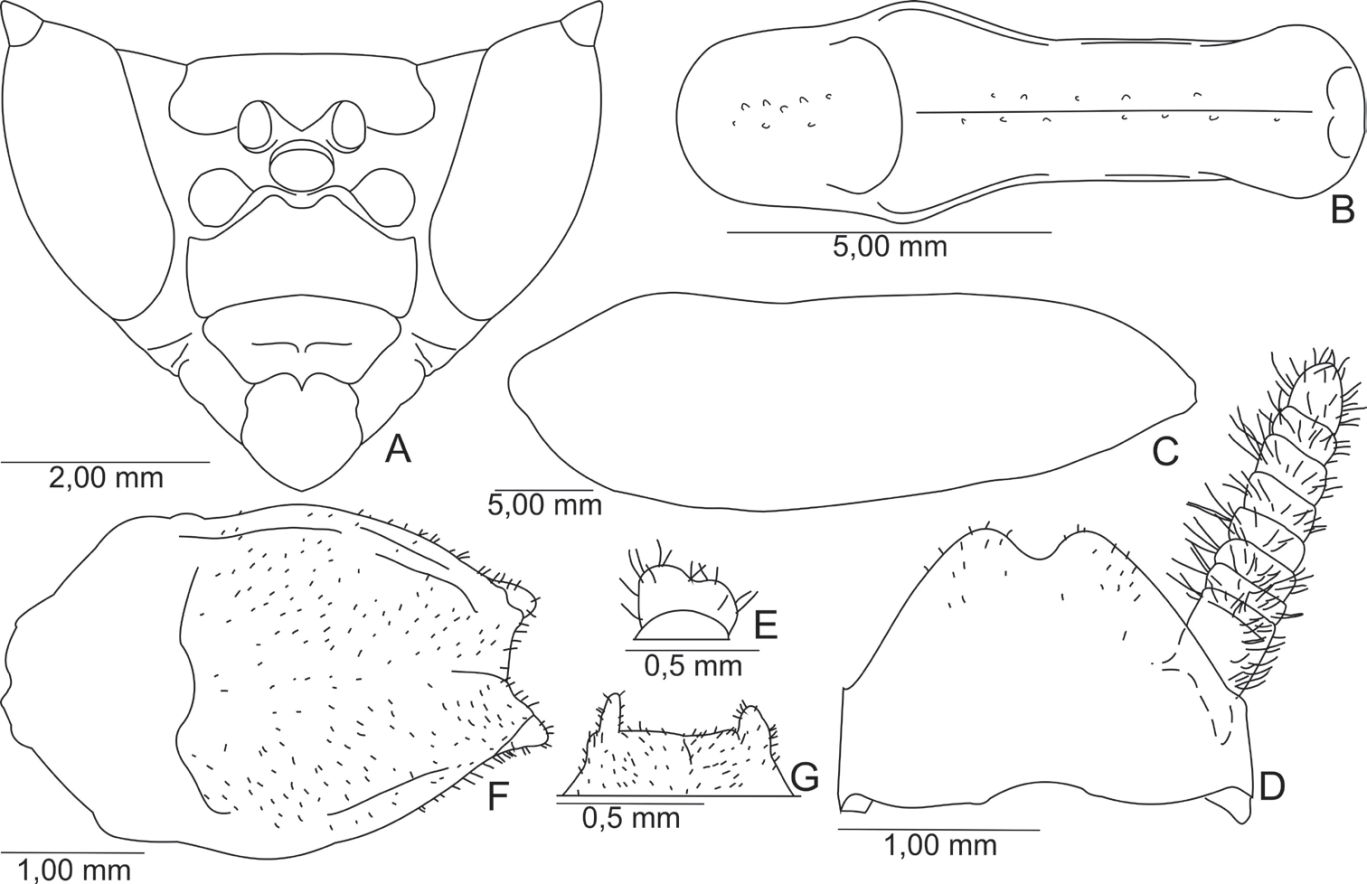
**A–D, F** holotype; **E, G** paratype. **A** Head, frontal view **B** Pronotum, dorsal view **C** Shape of the left mesothoracic wings, dorsal view **D** Supranal plate and cercus, dorsal view **E** Last cercomerum **F** Subgenital plate, ventral view **G** Distal margin of the subgenital plate, ventral view.

Head (Fig. 2A). Triangular, 1.67 times as wide as width of supracoxal dilatation. Antennae moniliform, brown, 1.07 times the length of the pronotum. Ocelli developed, elliptical. Vertex: rectilinear, below the imaginary line connecting top of compound eyes (not including the apical tubercle). Frontal shield transversal, 0.53 times wider than high.

Thorax. Pronotum, 0.27 times as long as length of body, 4.87 times longer than its smallest width, lateral margins smooth, surface with scattered small tubercles, distributed along sides of the medial carina (Fig. 2B). Prozona: anterior margin rounded, lateral margins slightly convergent. Metazona 2.05 times as long as length of prozona, with two basal flattened tubercles.

Fore legs. Coxae: stout, reaching base of proesternum, 0.75 as long as length of pronotum; anterior, posterior, and external margins with minute dispersed spines; posterior external face with small scattered tubercules, inner face with some circular ocher spots. Fore femora: stout, triangular, 0.94 times as long as the length of the pronotum; external face with small tubercules; 16 inner spines, except the spines of the genicular lobes; femoral spines of the three series black at tip. Fore tibiae: 0.55 as long as length of pronotum (not including the tibial claw); 20–21 external spines in left leg and 18–19 in right leg; 16 inner spines; external and inner tibial spines black at tips.

Mesothoracic wings: 3.29 times as long as length of pronotum, surpassing the abdomen at rest, and same length as posterior wings. Surface opaque and brown. Costal margin slightly concave and with small apical lobe (Fig. 2C). Venation brown. Venules of the costal area anastomosed.

Mid legs: pubescent; femora and tibiae 0.58 times as long as length of pronotum; first tarsomere shorter than length of all remaining tarsomeres.

Metathoracis wings: 3.03 times as long as length of pronotum, surface semi-hyaline; venation brown.

Hind legs: pubescent; femur 0.70 times as long as length of pronotum; tibiae 0.73 times as long as length of pronotum; first tarsomere shorter than length of all remaining tarsomeres.

Abdomen. Cylindrical; second to fourth and sixth tergite with distal black stripe, fifth tergite black; fourth and sixth tergite with rounded lateral lobe. Supranal plate: 1.47 times wider than length, distal margin bidentate (Fig. 2D). Cerci: bristly, cylindrical, eight cercomeri, last cercomerum cylindrical or bilobed (Fig. 2E) and slightly flattened. Subgenital plate: pubescent, oval (Fig. 2F). Styles: bristly, separated, small or more developed (Fig. 2G).

**Figure 3.**
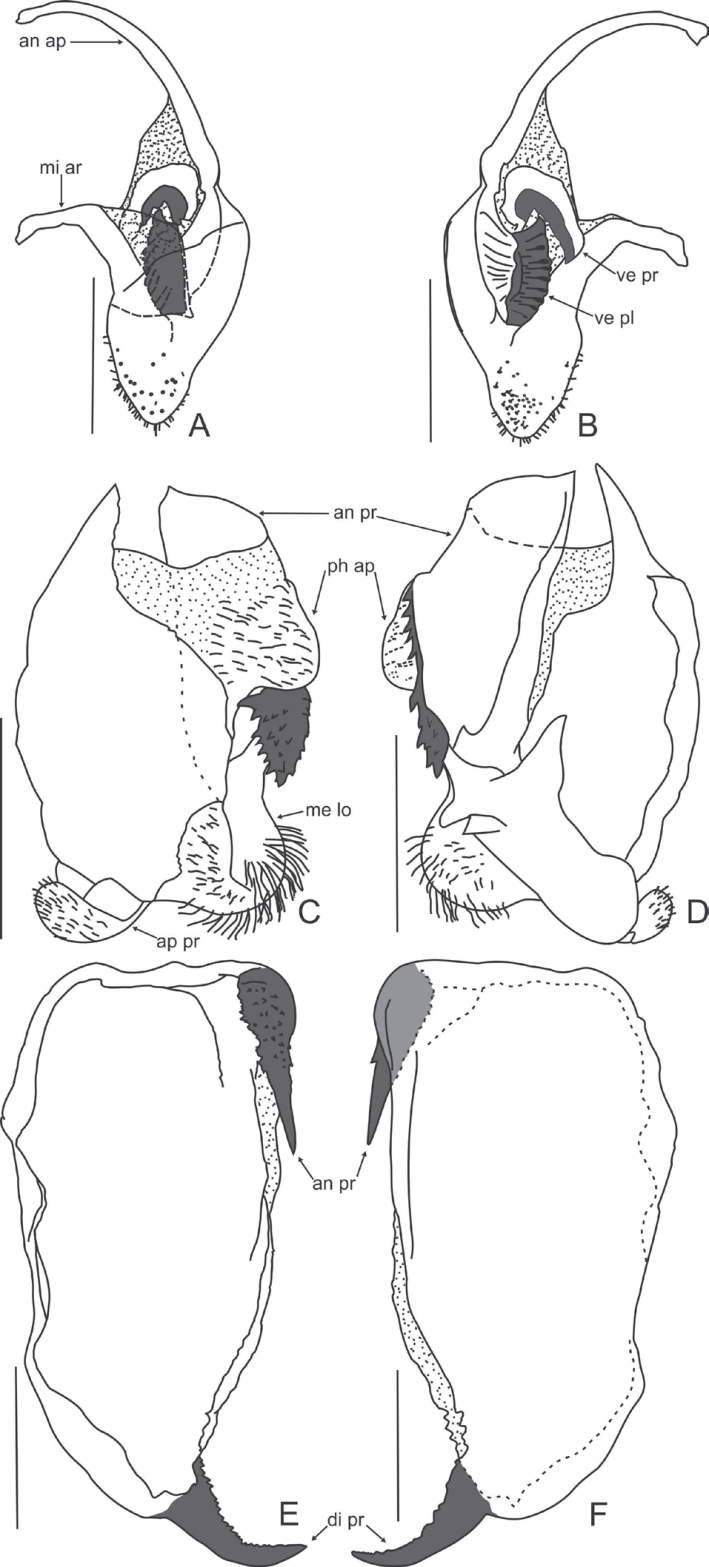
*Decimiana elliptica* sp. n., holotype. **A** Right dorsal phallomere, dorsal view **B** Right dorsal phallomere, ventral view **C** Left dorsal phallomere, dorsal view **D** Left dorsal phallomere, ventral view **E** Ventral phallomere, dorsal view **F** Ventral phallomere, ventral view. Abbreviations: **an ap** = anterior apodeme, **an pr** = anterior process, **di pr** = distal process, **me lo** = membranous lobe, **mi ar** = mid arm, **ph ap** = phalloid apophysis, **ve pl** = ventral plate, **ve pr** = ventral process. Scale bar = 1.00 mm.

Phallic complex. Right dorsal phallomere. Dorsal lamina triangular (Fig. 3A). Mid arm: developed, arched. Anterior apodeme long and narrow. Ventral plate sclerotized, well developed, trapezoidal, projected, with transverse grooves. Ventral process sclerotized, curved and well developed, as long as of ventral plate, forming acute angle backward (Fig. 3B).

Left dorsal phallomere. Dorsal lamina: ample, basal region narrow; right basal region membranous (Fig. 3C). Ventral lamina long and wide forming an anterior process, with distal sclerotized elliptical dentate lobe, connected to a lateral row of teeth which can be undeveloped (Fig. 3D). Apical process flattened, folded toward base of phallomere. Phalloid apophysis membranous, forming a relatively large and pilose lobe. Membranous lobe wide (approx. half the width of dorsal lamina), rounded and with long hair.

Ventral phallomere (Figs 3E, 3F). Elongated (aprrox. 1,84 times longer than wide). Tip of the right margin with well-sclerotized and acute anterior process, its surface covered with denticles. Distal process sclerotized, upward, with small denticles on anterior margin.

Female unknown.

#### Measurements

(mm). Holotype: body length 38.64, pronotum length 10.6, mesothoracic wings 34.93, metathoracic wings 32.2, fore coxae 8.0, fore femura 9.97, fore tibiae 5.91, mid femura 6.15, mid tibiae 6.15, hind femura 7.4, hind tibiae 7.82. Paratype: body length 42.68, pronotum length 11.71, mesothoracic wings 38.58, metathoracic wings 35.57, fore coxae 8.83, fore femura 11.01, fore tibiae 6.52, mid femura 6.79, mid tibiae 6.79, hind femura 8.17, hind tibiae 8.63.

#### Type localities.

**Figure 4.**
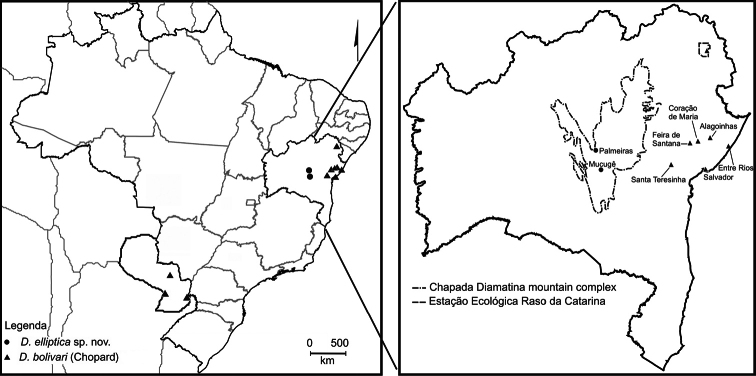
Geographical records of *Decimiana elliptica* sp. n. and *Decimiana bolivari* (Chopard).

The type specimens were collected in two localities in the Chapada Diamantina Mountain Range in Bahia State, northeastern Brazil: Parque Municipal de Mucugê (municipality of Mucugê) and near a mountain known as Morro do Pai Inácio (municipality of Palmeiras) (Fig. 4). The Chapada Diamantina represents the northern portion of the Espinhaço Range (Rocha et al. 2005) and according Velloso et al. (2002) it is considered an eco-region of the Caatinga (dryland) Biome, with a rainy season generally from November to April. The vegetation of the Chapada Diamantina is a mosaic of “caatinga”, “cerrado de altitude”, “campos rupestres”, and semideciduous and deciduous forests.

## Discussion

Regarding the right dorsal phallomere is only possible to compare with *Decimiana bolivari*, accurately illustrated by Heitzmann-Fontenelle (1964) Fig. 5. Fact that we attest by confrontation with the material examined of *Decimiana bolivari*. Since the literature uses the lack of description and illustration of this phallomere in the other species of genus.

The ventral lamina in the right dorsal phallomere in *Decimiana elliptica* sp. n. is grooved while in *Decimiana bolivari* is covered by spines. The ventral process in the new species is more curved in the base and more elongated than in *Decimiana bolivari*.

The anterior process of the left dorsal phallomere is developed and anteroposteriorly perpendicular to phallomerein *Decimiana elliptica* sp. n., *Decimiana bolivari*, *Decimiana herbardi*, *Decimiana rehni* and *Decimiana tessellata* (Figs 3C, 3D; Heitzmann-Fontenelle 1964: Fig. 6; Lombardo 2000: Figs 27–29,31), and . differs from *Decimiana clavata* because the anterior process is less developed and oblique (Ippolito and Lombardo 2004: Figs 4, 5). In the new species the anterior process of the left dorsal phallomere has truncated shape, while *Decimiana bolivari*, *Decimiana herbardi*, *Decimiana rehni* it is sack-shaped and in *Decimiana tesselata* has an expanded apex.

On the surface of the anterior process of the left dorsal phallomere are absent in *Decimiana elliptica* sp. n. the minute spines that are present in *Decimiana bolivari* and *Decimiana herbardi*, as well as, are absent in the new species the teeths present in *Decimiana rehni* and the large apical teeths observed in *Decimiana tesselata*.

The anterior process of the left dorsal phallomere in *Decimiana elliptica* sp. n. and *Decimiana clavata* has a distal esclerotized lobe, which it is absent in D.* bolivari*, *Decimiana herbardi*, *Decimiana rehni* and *Decimiana tessellata*. However, this sclerotized lobe is elliptical in *Decimiana elliptica* sp. n. and bludgeon-shaped in *Decimiana clavata*.

The membranous lobe of the left dorsal phallomere the new species is relatively greater and more rounded than the other species, except for *Decimiana clavata* which it is most similar.

*Decimiana elliptica* sp. n. has the ventral phallomere more elongated than the other species (Fig. 3E). The anterior process of the ventral phallomere elongated as in *Decimiana bolivari* (Heitzmann-Fontenelle 1964: Fig. 2), *Decimiana rehni* (Lombardo 2000: Fig. 25) and *Decimiana herbardi* (Lombardo 2000: Fig. 26). The distal process of the ventral phallomere of new species resembles that of *Decimiana bolivari* in the form, curved upwards. However differs the distal process of *Decimiana tessellata* who is backwards, and the distal process of *Decimiana rehni* which is more curved and short.

### Notes on the distribution of *Decimiana bolivari* (Chopard, 1916)

Material examined: BRASIL, Bahia: Alagoinhas, 24.X.1993, D. H. Smith, col., male (UFBA). Coração de Maria, Distrito de Retiro, 07.XI.2010, Franklin Machado col., male (MZUEFS #53183); *ib*., female (MZUEFS #53180). Entre Rios, 07.V.2008, Silva-Neto, A. col., male (MZUEFS #39218). Feira de Santana, 16.XII.1999, Márcia col., male (MZUEFS #13268); *ib*., 14.V.2001, Ivan Castro col., male (MZUEFS #13269); *ib*., 08.XI.2003, F. Bravo col., male (MZUEFS #13285). Salvador, 23.IV.1985, D. H. Smith, col., male (UFBA); *ib*., 2.X.1952, Afonso Braga, col., 1 male (FIOCRUZ); *ib*., 13.V.1962, Ivo Silva col., male (FIOCRUZ). Santa Teresinha, Serra da Jibóia, ca. 800 m a.s.l., VIII.2004, Raimunda col., male (MZUEFS #28123); *ib*., 29.III.2009, male (MZUEFS #46032). Estação Ecológica Raso da Catarina, 14.VI.1981, D. H. Smith, col., male (UFBA).

In his review of *Decimiana*, Lombardo (2000) stated that *Decimiana bolivari* was only present in Paraguay (known records from Asunción, “central Paraguay”, Horqueta, Puerto Bertoni, Puerto San Pablo y Sapucay, *sensu* Lombardo 2000); however, he did not comment on the Brazilian records made by Terra (1995), who reported (without mentioning specific localities) that *Decimiana bolivari* occurs in the Brazilian states of Mato Grosso do Sul and Bahia. He also no mention Heitzmann-Fontenelle (1964) that record *Decimiana bolivari* in state of Mato Grosso do Sul, Serra do Urucum. Because of the lack of references to specific material, it was impossible to locate the specimens studied by Terra (1995) and thus these records cannot be confirmed. Ehrmann (2002) and Agudelo et al. (2007) followed the distributions of *Decimiana bolivari* presented by Lombardo (2000).

However, we confirm herein the occurrence of *Decimiana bolivari* in Bahia state for the following localities: Alagoinhas, Coração de Maria, Entre Rios, Feira de Santana, Salvador, Santa Teresinha (Serra da Jibóia) and Estação Ecológica Raso da Catarina (Fig. 4). These confirmed records considerably expand the distribution of *Decimiana bolivari*, to the Brazilian northeast.

It is important to mention that the specimens of *Decimiana bolivari* from Bahia were collected in various ecosystems encompassing a wide array of different climates and plant formation. This species was recorded from localities in ombrofilous Atlantic Rain Forest with a moist climate (Entre Rios and Salvador), in transitional ecosystems between Caatinga (dryland) and Atlantic Rain forests (Alagoinhas, Coração de Maria, Feira de Santana, Santa Teresinha) with semi-arid to moist climates, and in semideciduous forest/caatinga and semi-arid caatinga/deciduous forest (Raso da Catarina Ecological Station) (Velloso et al. 2002, IBGE 2011, SEI 2011). *Decimiana bolivari* was also collected at sea level (e.g. Entre Rios and Salvador) and up to approximately 800 (Santa Teresinha, Serra da Jibóia) (IBGE 2011, SEI 2011, collection data). and Pantanal region, domained. In Mato Grosso do Sul state (Serra do Urucum), *Decimiana bolivari* is recorded in the Pantanal ecosystem, where occurs the plant formations: steppic savanna/seasonal forest; savanna/steppic savanna and steppic savanna grass-woody (IBGE 2004).

*Decimiana bolivari* seems to be a species well adapted to different biotic, environmental, and altitudinal conditions, further collections will provide a additional insights on the actual extent of its distribution.

## Supplementary Material

XML Treatment for Decimiana elliptica
